# Safety and tolerability of an ovine-derived polyclonal anti-TNFα Fab fragment (AZD9773) in patients with severe sepsis

**DOI:** 10.1186/cc9683

**Published:** 2011-03-11

**Authors:** P Morris, B Zeno, A Bernard, X Huang, S Simonson, G Bernard

**Affiliations:** 1 Wake Forest University School of Medicine, Winston Salem, NC, USA; 2 Riverside Methodist Hospital, Columbus, OH, USA; 3 University of Kentucky, Lexington, KY, USA; 4 AstraZeneca Charnwood, Loughborough, UK; 5 AstraZeneca, Wilmington, DE, USA; 6 Vanderbilt University, Nashville, TN, USA

## Introduction

Sepsis remains a significant medical problem. TNFα is a central cytokine in sepsis pathophysiology. We conducted a phase IIa trial in patients with severe sepsis to assess the safety and tolerability of an intravenously infused ovine-derived polyclonal anti-TNFα Fab fragment (AZD9773).

## Methods

This was a double-blind, placebo-controlled, dose-escalation trial (NCT00615017) with 2:1 randomisation (active:placebo). Two single-dose cohorts (50 units/kg and 250 units/kg) and three multiple-dose cohorts (250 units/kg followed by nine doses of 50 units/kg every 12 hours, 500 units/kg followed by nine doses of 100 units/kg, 750 units/kg followed by nine doses of 250 units/kg) were studied. Safety was assessed by monitoring adverse events (AEs), mortality, and laboratory safety measures, including formation of human anti-sheep antibodies (HASA) and their association with AEs.

## Results

A total of 70 patients were studied. The mean age was 56 years, 46% were male, and the mean APACHE II score was 26. About 50% of patients had two organ failures (both respiratory and cardiovascular). Multiple doses of AZD9773 reduced circulating TNFα towards the limit of detection in most patients throughout the 5 days of dosing. The most common serious AEs were mainly related to the underlying illness and included: sepsis, pneumonia, septic shock and respiratory failure across all groups. Table 1 summarises the safety outcomes. Development of HASA did not appear to be associated with either decreased TNFα reduction or specific AEs.

**Table 1:** Safety outcomes with AZD9773 administration

	Single-dose cohorts combined (*n *= 17)	Multiple-dose cohorts combined (*n *= 30)	Placebo (*n *= 23)
Mortality, *n *(%)	6 (35%)	7 (23%)	6 (26%)
Any treatment-emergent AEs	17 (100%)	27 (90%)	23 (100%)
Treatment-emergent AEs related to study drug	2 (12%)	7 (23%)	10 (43%)
Patients with any serious AEs	9 (53%)	14 (47%)	13 (57%)

## Conclusions

Administration of AZD9773 in patients with severe sepsis reduced circulating TNFα levels and had a safety profile similar to placebo administration. A larger randomised phase IIb clinical trial (NCT01145560) is ongoing to further characterise the safety and efficacy of AZD9773 in patients with severe sepsis.

